# High level of heterozygous haplotype of hemoglobin in Abidjan population with mild malaria

**DOI:** 10.1186/s12920-022-01263-3

**Published:** 2022-05-23

**Authors:** Tosséa A. Stéphane Koui, Alloh Albert Gnondjui, Adji Eric Gbessi, Ako Aristide Bérenger Ako, Baba Coulibaly, A. Delpêche Aka, Bi Sery E. Gonedele, Offiana André Toure, Ronan Jambou

**Affiliations:** 1 Biosciences Department, Université Félix Houphouët-Boigny de Cocody, Abidjan, Côte d’Ivoire; 2 Department of Parasitology-Mycology, Institut Pasteur de Côte d’Ivoire, Abidjan, Côte d’Ivoire; 3 Global Health Department, Institut Pasteur, 25 rue Dr Roux, 75015 Paris, France; 4 CERMES Niger, Niamey, Niger

**Keywords:** Sickle cell anemia, Haplotype, Ivory coast, Malaria

## Abstract

**Background:**

Sickle cell disease (SCD) is a hemoglobin disorders that concern 300,000 newborns each year around the world. There are hemoglobin haplotypes that affect SCD clinic expression.

**Methods:**

Our goal was to identify the hemoglobin’s haplotypes among individuals with mild malaria independently of SCD status in Côte d’Ivoire. To determine these haplotypes, specific restriction enzyme (RE) is used after PCR amplification with each primer. According to the digestion of PCR product by RE, five hemoglobin’s haplotypes are found in the world.

**Results:**

In Côte d’Ivoire, no study has yet deeply described the distribution of haplotypes. Four different “classical” haplotypes of hemoglobin were detected: Benin (56.5%), Bantou (28.5%), Senegal (4%), Cameroun (1%); and 10% of atypical profiles. Heterozygous haplotype (69%) were more frequent than homozygous haplotype (31%).

**Conclusions:**

In this preliminary study, we note a high prevalence of atypical and heterozygous haplotype. Benin haplotype that is associated with severity of SCD was most predominant in our studied population.

## Background

Sickle cell disease (SCD) is a hemoglobin disorders that concerns 300,000 newborns each year around the world [1]. Sub-Saharan’s countries harbored the highest prevalence with S hemoglobin in Central Africa and C in Sahelian areas [2–4]. There are hemoglobin haplotypes that affect SCD clinic expression. These haplotypes have different origins [5, 6] and some studies indicated the relation between SCD clinics manifestations and hemoglobin haplotypes. In fact, the presence of some haplotypes reduce the SCD symptoms; and other haplotypes amplify the clinics manifestations [7–14]. To determine these haplotypes, specific restriction enzymes (RE) are used after PCR amplification. Relative to the digestion of PCR product by RE; there are five hemoglobin’s haplotypes around the world: Bantou (CAR), Arabic, Senegal, Cameroun and Benin [15, 16]. In Côte d’Ivoire, no study has yet deeply described the distribution SCD haplotypes. Here we conducted a retrospective preliminary study in Côte d’Ivoire to identify the hemoglobin’s haplotypes among individuals with mild malaria independently to SCD status.

## Materials and methods

### Sample collections

Abidjan is the economic capital of Côte d’Ivoire with five million inhabitants from a large number of neighboring countries. Participants were recruited from CSUCOM Anonkoua-Kouté (Abidjan, Abobo) in 2013 and 2016 among patients attending the center with mild malaria (parasitemia more than 2000 parasites/µL blood).

The hemoglobin status of the patients was determined based on a standard acetate electrophoresis of hemoglobin using *Sebia*^*®*^* Hemoglobin electrophoresis*, following the protocol of the manufacturer.For molecular typing, 50 µL of total blood was dried on *5 M Whatman*^*®*^ paper and stored in zip locked bag contained silicate gel until use in 2020.

### Haplotype molecular typing

The hemoglobin electrophoresis was performed following the manufacturer’s recommendations with total blood. For molecular typing, DNA purification was performed on blood spots using the *Qiagen*^*®*^*Blood Minikit* as recommended by the manufacturer. For amplification, different programs were used according to the couple of primers used. Nine pairs of primers have been used following Sutton and co [15] and Doupa and co [17] (Table 1). After amplification, each type of PCR products was digested with a related restriction enzyme (RE). The haplotype profiles were identified according to Sutton and co [15] and Doupa and co [17]. (Table 2).

**Table 1:** Primers used in this study

Primer name	Primer sequence	Source
5’ Gγ	AACTGTTGCTTTATAGGATTT T	[17]
	AGGAGCTTATTGATAACCTCAGAC	
Gγ	TGCTGCTAATGCTTCATTACA A	
	AAGTGTGGAGTGTGCACATGA	
Aγ	TGCTGCTAATGCTTCATTACA A	
	TAA ATGAGGAGCATGCACACA C	
Ψβ	GAA CAG AAG TTG AGA TAG AGA	
	ACT CAG TGG TCT TGT GGG CT	
3’Ψβ	TCT GCA TTT GAC TCT GTT AGC	
	GGA CCC TAA CTG ATA TAA CTA	
3’ δ	TGG ATT CTG CCT AAT AAA A	
	GGG CCT ATG ACA GGG TAA T	
Β	GCT GAG GGT TTG AAG TCC AA	[15]
	CAC TGA TGC AAT CAT TCG TC	
5' β	CTACGCTGACCTCATAAATG	
	CTAATCTGCAAGAGTGTCT	
3' β	TTCATACATAACAATACTCA	
	GAGGAGAGCTTTACTTCCAA

**Table 2:** Hemoglobin Haplotypes restriction profiles described and in this study

	Gene and restriction enzyme								
	5gamma (XmnI)	Gamma (HindIII)	Alpha (HindIII)	phiBêta (HincII)	3phiBêta (HincII)	3Delta (HinfI)	Beta (AvaII)	5Beta (HinfI)	3Beta (HpaI)
*Haplotype described* [2, 3]									
Senegal	+	+	−	+	+	+	+	+	+
Benin	−	−	−	−	+	−	+	+	−
Bantu (CAR)	−	+	−	−	−	−	+	+	+
Cameroon	−	+	+	−	+	+	+	−	+
Arabic	+	+	−	+	+	−	+	−	+
*Atypic in this study With Bantu haplotype*									
Atypic 1(1) *	−	−	−	−	+	+	+	+	+
*With Benin haplotype*									
Atypic 1(4) *	−	−	−	−	+	+	+	+	+
Atypic 2(6) *	−	+	−	−	+	+	+	+	+
Atypic 3(4) *	−	+	−	−	+	−	+	+	+
Atypic 4(1) *	−	−	−	−	+	−	+	+	+
*Only atypic*									
Atypic 2(4) *	−	+	−	−	+	+	+	+	+

** + ** = Presence of restriction enzyme site / − = Absence of restriction enzyme site

*CAR* Central African Republic

*() Proportion of allele, N = 20 atypics alleles

## Results

### Demographic results

Of the total 100 patients included in the study, 55% were women. The average age of the patients recruited was 14.5 years.

### Haplotype typing

**Fig. 1:**
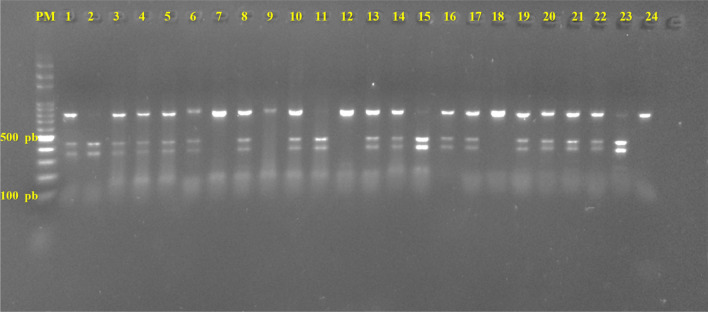
Gamma PCR products digestion by HindIII (Restriction Enzyme). PCR product size for Gamma is 782 pb xhich gives two fragments of 436 pb and 346 pb. After digestion by HindIII. Homozygote sample will present only 782 pb lane or 436 pb and 346 pb. Heterozygote sample will present three (03) lanes: 782 pb, 436 pb and 346 pb. On this gel, for example, homozygotes samples without restriction site of HindIII are N°7, N°9, N°12, N°18 and N°24. Homozygotes with restriction site are N°2, N°11, N°15 and N°23. All other are heterozygotes

Four different “classical” haplotypes of hemoglobin were detected, Benin (56.5%), Bantou (28.5%), Senegal (4%) and Cameroun (1%). The Arabic haplotype was not observed. In addition, 10% of atypical profiles were detected (i.e. 20 haplotypes). Atypic haplotypes were presents in the groups of Benin (15/20) and Bantou (01/20) (Table 2, Fig. 1).

Heterozygous (69%) were more frequent than homozygous (31%). For homozygote, women were more affected than men (40/69 and 29/69 respectively).

### Hemoglobin typing and malaria diagnostic

The AA genotype represented 87% of the samples. The other genotypes were AC, AS, SC and CC (8%, 2%, 2% and 1% respectively, Table 3).

**Table 3:** Distribution of haplotype according malaria and hemoglobin typing

	Mean (parasitaemia tpz/µL blood)	Hemoglobin typing (N*)					Total (N*)
		AA	AC	AS	CC	SC	
*With Benin haplotype*							
Benin/Atypic	44,644	14	1				15
Benin/Benin	40,342	20	1		1	1	23
Cameroon/Benin	43,768	2					2
Senegal/Benin	31,017	6	1				7
		42	3		1	1	47
*With Bantu haplotype*							
Atypic/Bantu	84,307	1					1
Bantu/Bantu	75,460	5		1			6
Senegal/Bantu	21,866	1					1
		7		1			8
*With Bantu and Benin haplotypes*							
Bantou/Benin	48,930	36	5	1		1	43
*Another haplotype*							
Atypic/Atypic	36,279	2					2
Total	46,376**	87	8	2	1	2	100

Data are available at https://ega-archive.org/studies/EGAS00001006008

*N: Proportion of participants; **Mean of parasitaemia in this study

Mean parasitemia was 46,376 parasites/µL blood, without any significant difference between haplotypes (*t*-test, *p* = 0.95).

All participants with AC or CC genotypes were from the Benin group (homozygote Benin/Benin or Benin/Bantu) whereas AS was found in the Bantu group (Table 3). Atypic, Cameroun and Senegal haplotypes were observed only in normal hemoglobin group (Table 3).

## Discussion

Several authors have highlighted the interest of studying hemoglobin haplotypes for individuals with hemoglobin disorders as a modulation of the clinical profile of the disease [7–14]. In Côte d'Ivoire, there is not available data on hemoglobin haplotypes. This study updates data on hemoglobin haplotypes in Côte d'Ivoire amongst individuals living in Abidjan and experimenting mild malaria. During this work, women represented the highest proportion of people attending dispensaries. This is frequently observed as men use to practice self-treatment so they rarely visit dispensaries. Our study indicated a prevalence of 13% of sickle cell trait (3% of SCD) in the population analyzed. Previous studies conducted by Tossea et al*.* [18] in the same area reported a similar prevalence. This concordance could be due to the design of the two (02) studies. Indeed, these studies were carried out in individuals with middle malaria in Abidjan.

The Benin haplotype was the most prevalent followed by the Bantu one, which can be attributable respectively to ethnic origin of the population in Abidjan, and the high level of migration from central Africa to Ivory Coast. Arab-Indian haplotype was not observed despite migration of populations across the Sahel (Peul and Toucouleur ethnics). A small prevalence of Senegal and Cameroon haplotypes was observed. These different prevalence are in accordance with the geographical distribution of the different populations [5, 6].

Similar to the studies of several authors who reported 5–10% of atypical haplotypes [9, 19–21], we found 10% of atypical haplotypes in the population of Abidjan, mostly associated with the Benin haplotype. Due to the fact that the Benin haplotype is associated with a more severe form of expression of SCD and considering the high prevalence of atypical haplotypes, the relationship between these haplotypes and the clinical pattern of sickle cell disease should be investigate further.

Overall a high proportion of heterozygous genotype (61%) was found in Abidjan. That differs from the studies conducted by Doupa et al. (32%) [17]. This difference could be due to their selection of patients harboring SCD and to the limitation of that study to only a single restriction site.

The emergence of these atypics haplotypes and the high proportion of heterozygous haplotypes could support a high level of mixed populations. Indeed, Abidjan is one of the major city in West Africa, with a cosmopolite population. In addition, the important mixing of populations would be a factor in the development of genetic phenomena such as chromosomal recombination’s between haplotype.

Different atypical haplotypes observed show strong similarities with the Benin and Bantu haplotypes. Several others authors [19, 22, 23] obtained similar results relative to atypical haplotypes. The high proportion of atypical haplotype and its similarity with the Benin haplotype could be explained by selection pressure. Indeed, the association of the atypical haplotypes with the more severe Benin haplotype may lead to a more moderate expressive expression of sickle cell disease. More insight studies need to be conducted to explore such associations and their clinic expressions.

## Conclusion

In this preliminary study, we note a high prevalence of atypical and heterozygous haplotype. Benin haplotype that is associated with severity of SCD was most predominant in our studied population. Further studies involving a large number of SCD participants could help to estimate an accurate prevalence of hemoglobin haplotypes in Côte d’Ivoire.

## Data Availability

The datasets used and/or analysed during the current study are available from the corresponding author on reasonable request.

## Abbreviations

SCD: Sickle cell disease
RE: Restriction enzyme
PCR: Polymerase chain reaction
